# The Homeobox Only Protein Homeobox (HOPX) and Colorectal Cancer

**DOI:** 10.3390/ijms141223231

**Published:** 2013-11-25

**Authors:** Keishi Yamashita, Hiroshi Katoh, Masahiko Watanabe

**Affiliations:** Department of Surgery, Kitasato University School of Medicine, Asamizodai 2-1-1, Minami-ku, Sagamihara, Kanagawa 252-0374, Japan; E-Mails: mohiro-kato@hotmail.co.jp (H.K.); gekaw@med.kitasato-u.ac.jp (M.W.)

**Keywords:** HOPX, colorectal cancer, methylation, tumor suppressor gene

## Abstract

The HOP (homeobox only protein) homeobox (HOPX) is most closely related to the homeobox protein that contains a homeobox-like domain but lacks certain conserved residues required for DNA binding. Here, we review the current understanding of HOPX in the progression of colorectal cancer (CRC). HOPX was initially reported as a differentiation marker and is expressed in various normal tissues. In the colon, HOPX is expressed uniquely in the quiescent stem cell, +4, and in differentiated mucosal cells of the colon. HOPX expression is markedly suppressed in a subset of cancers, mainly in an epigenetic manner. CRC may include separate entities which are differentially characterized by HOPX expression from a prognostic point of view. HOPX itself can regulate epigenetics, and defective expression of HOPX can result in loss of tumor suppressive function and differentiation phenotype. These findings indicate that HOPX may be both a central regulator of epigenetic dynamics and a critical determinant for differentiation in human cells. HOPX downstream targets were identified in CRC cell lines and hold promise as candidates for therapeutic targets of CRC, such as EphA2 or AP-1. Further analysis will elucidate and confirm the precise role of such proteins in CRC progression.

## 1. Introduction

**Figure 1.**
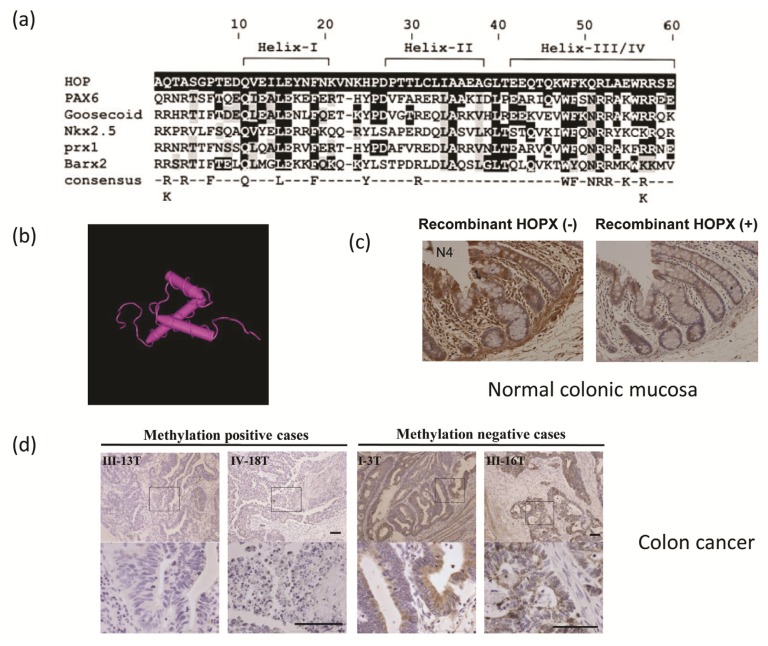
(**a**) Sequence comparison of the homeodomain of mouse homeobox only protein (HOP) with other homeodomains. Amino acid positions within the 60 amino acid homeodomain are shown; (**b**) Solution Structure of the Homeodomain-only Protein HOP from NCBI structure summery; (**c**) HOP homeobox (HOPX) expression in the normal colonic mucosa (**left**). HOPX peptide is absorbed to eliminate non-specific staining of HOPX (**right**); (**d**) HOPX expression was suppressed in colon cancer with promoter DNA hypermethylation, while its expression was sustained in colon cancer with no methylation. Scale bar, 100 μm.

HOP (homeobox only protein) homeobox (HOPX) was identified in mouse and humans by searching an EST database using the PAX3 homeobox as a probe [1]. The human and mouse HOPX proteins contain 73 amino acids, including a 60 amino acid motif homologous to HOX proteins, and share 92% identity. The mouse and human HOPX proteins are most closely related to the homeobox protein HOX6 and goosecoid, sharing approximately 40% identity within the homeobox-like domain (Figure 1a). HOPX lacks certain conserved residues required for DNA binding. In mouse, *HOPX* gene expression is initiated early in cardiogenesis and continues in cardiomyocytes throughout embryonic and postnatal development. Northern blot analysis of adult and embryonic mouse tissues detected a 1.2-kb transcript in embryonic and adult heart and in adult brain, lung, liver, intestine, and spleen [1]. Shin *et al*. independently cloned mouse *HOPX* and identified the human homolog [2]. They determined that HOPX forms three alpha helices which fold into a helix-turn-helix motif characteristic of the homeobox (Figure 1b).

HOPX is highly expressed in the developing heart, where its expression is dependent on the cardiac-restricted homeobox protein, Nkx2.5. Genetic and biochemical data indicate that mouse HOPX functions directly downstream of Nkx2.5, and that HOPX physically interacts with serum responsive factor (SRF) and inhibits SRF-dependent transcription by inhibiting SRF binding to DNA [1]. Shin *et al*. confirmed that mouse HOPX does not bind DNA and acts as an antagonist of SRF, which regulates the opposing processes of proliferation and myogenesis [2]. Kook *et al*. also showed that HOPX can inhibit SRF-dependent transcriptional activation by recruiting histone deacetylase (HDAC) activity and can form a complex that includes HDAC2 [3,4]. Transgenic mice overexpressing HOPX develop severe cardiac hypertrophy, cardiac fibrosis, and die prematurely. A mutant form of HOPX, which does not recruit HDAC activity, did not induce hypertrophy. Kook *et al*. therefore concluded that chromatin remodeling and repression of active transcriptional processes can result in hypertrophy and heart failure; intriguingly, this process can be blocked with chemical HDAC inhibitors.

**Figure 2.**
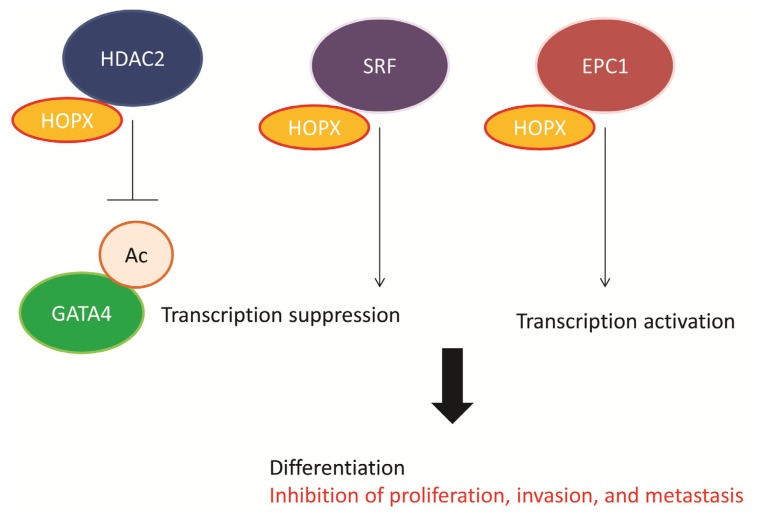
Model depicting Hdac2 interacting with Hopx to induce deacetylation of Gata4 and modulation of cell cycle genes (**left**); Model depicting serum response factor (SRF) interacting with Hopx to suppress SRF transcriptional activity and modulation of growth related genes (**middle**); Model depicting enhancer of polycomb 1 (EPC1) interacting with Hopx to induce differentiation related genes (**right**).

Kee *et al*. used yeast 2-hybrid analysis to identify enhancer of polycomb 1 (EPC1), which interacts with HOPX. Expression of EPC1 was upregulated during differentiation of a rat myoblast cell line into skeletal myocytes [5]. Differentiation was induced by EPC1 overexpression, and was severely impaired in EPC1-knockdown cells. Cotransfection of HOPX potentiated EPC1-induced transactivation of myogenin and myotube formation. Kee *et al*. concluded that EPC1 plays a role in initiation of skeletal muscle differentiation and that its interaction with HOPX is required for full activity. Hence, the function of HOPX is to regulate epigenetics (Figure 2).

HOPX has been reported to play a critical role in the differentiation of various cells such as trophoblasts [6,7], keratinocytes [8,9], T cells [10,11] and lung alveolar cells [12]. Most intriguingly, HOPX is strongly expressed in quiescent colon stem cells [13] and hair follicle cells [14].

## 2. Genomic Structure of *HOPX* Gene

**Figure 3.**
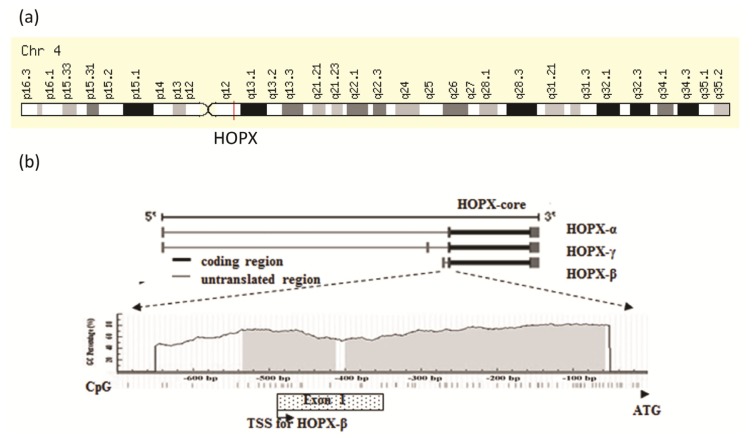
(**a**) Chromosomal location of *HOPX*; (**b**) Schematic diagram of the three spliced transcript variants and a common transcript core in *HOPX* (**top**) and of CpG islands (dark areas) in the 5′-flanking region of *HOPX* gene (**bottom**). The only HOPX-β harbors CpG islands encompassing the first exon and intron. Vertical bars indicate the dinucleotide CpGs.

The International Radiation Hybrid Mapping Consortium mapped the *HOPX* gene to chromosome 4 (Figure 3a). Previous studies using comparative genomic hybridization have reported copy number loss at this region in hepatocellular carcinoma [15,16]. Loss of heterozygosity (LOH) at 4q12–q13 was also identified in breast [17] and hepatocellular carcinomas [18]. LOH analysis was performed using three microsatellite markers, D4S189, D4S231 and D4S392, around the region of chromosome 4q12 in 29 paired primary lung tumor samples, where HOP locates. LOH was found in 4 out of 23 cases (17.4%) [19]. These findings suggest the existence of a tumor suppressor gene at 4q11–q13, with HOPX being one of the candidate genes. Although HOPX has three spliced variants, the coded proteins are identical (HOP core). The location of CpGs in the *HOPX* genomic sequence is shown in Figure 3b[20]. Of the three spliced transcript variants, only HOPX-β promoter harbors CpG islands encompassing the first exon and intron, whereas the same promoter for HOPX-α and HOPX-γ does not harbor any CpG islands near the transcription start site.

## 3. Colorectal Cancer (CRC) and *HOPX* Gene Expression

HOPX is strongly expressed in normal colorectal mucosa tissues, and this Immunohistochemical staining is absorbed by HOPX peptide pretreatment, as previously reported [21] (Figure 1c). On the other hand, HOPX expression is dramatically downregulated in subsets of primary CRC (Figure 1d). Recent examination of cancer genetics data revealed that down-regulation of important tumor suppressor genes is often caused by DNA hypermethylation of promoter CpG islands in a cancer-specific manner. *HOPX* is one of these tabulated genes [20,22–24]. Specifically, the expression of HOPX is suppressed in various primary cancer tissues [12,20,21,23,25–29] (Table 1). Among these cancers, DNA methylation is prevalent in esophagus [23], stomach [20], and pancreas [21] cancers, as well as colorectal cancer (CRC) [26].

CRC is a well-studied cancer in which both gene expression and DNA methylation status have been examined. In CRC, HOPX methylation status reflects HOPX expression levels well [26]. Specifically, primary CRC tissues or colorectal mucosa tissues with HOPX hypermethylation show a low level of HOPX expression, and vice versa. More intriguingly, the demethylating agent 5-aza-2′-deoxycytidine, and/or the HDAC inhibitor trichostatin A, robustly reactivated HOPX expression. Reactivation was especially pronounced for HOPX-β, which harbors CpG islands in the CRC cell lines, DLD1 and HCT116. These findings suggest that HOPX expression is regulated mainly by epigenetic control in CRC.

Quantitative TaqMan MSP (methylation specific PCR) showed significantly higher methylation levels in primary CRC tissues than in the corresponding normal mucosal tissues [26]. This analysis further elucidated that the HOPX methylation value significantly increases during progression from non-metastatic disease (pN0) to metastatic disease (pN1) in CRC. Even in patients with primary CRC and positive lymph nodes (Stage III), patients with higher HOPX methylation showed poorer prognosis than those with lower HOPX methylation. These findings suggest that HOPX hypermethylation represents an aggressive phenotype of CRC.

On the other hand, poorly differentiated CRC is relatively rare among CRC, and is characterized by dismal prognosis as compared with differentiated CRC. It is intriguing that poorly differentiated CRC harbors more highly hypermethylated HOPX than differentiated CRC [26,30].

## 4. Functional Role of HOPX in CRC

Cancer-specific hypermethylation of CpG islands of the promoter regions suggests that such genes have tumor suppressor function [22,31,32]. HOPX has been demonstrated to have tumor suppressor function in various cancers, including CRC (Table 2). Asanoma *et al*. (2003) transfected HOPX into choriocarcinoma cell lines and observed remarkable alterations in cell morphology and suppression of *in vivo* tumorigenesis [28]. *In vitro* or *in vivo* tumorigenesis has been repeatedly proven to be suppressed by *HOPX* gene transfection in esophageal cancer [23], endometrial cancer [29], gastric cancer [20], and pancreatic cancer [21]. In the CRC cell lines, DLD1 and HCT116, HOPX transfection strongly suppressed tumorigenesis in nude mice and in a soft agar assay [26]. In DLD1 transfected with HOPX, stromal angiogenesis on day 15 was remarkably suppressed. Moreover, TUNEL assay was performed in tumors derived from HOPX or mock transfected DLD1, and apoptotic cells were significantly more recognized in HOPX transfected cells than in mock cells. Cell cycle analysis showed that *HOPX* transfection increased the subG1 population and the level of caspase-3 is higher in HOPX-transfected cells than in mock cells. These findings strongly indicate that *HOPX* is a tumor suppressor gene in CRC.

HOPX may be involved in cell invasiveness in CRC and other cancers [12,20,21]. Matrigel invasion assays of CRC cell lines showed that HOPX transfection results in a remarkable reduction of invaded cells [26]. F-actin labeling with Phalloidin revealed that the mock cells exhibited active filopodia, whereas HOPX-expressing cells exhibited fewer filopodia fibers and showed F-actin aggregated in the cytoplasm. Recent reports studying other cancers demonstrated the mechanism of augmented cancer invasion in HOPX knockdown cells; for example, HOPX knockdown is involved in marked phosphorylation of FAK (focal adhesion kinase) and integrin α5 in human lung cancer [12]. In a recent report on sarcoma cells, down-regulation of the *HOPX* gene unexpectedly decreased metastatic activity and identified genes associated with metastasis, such as integrin α 4 [33]. Given the prognostic relevance of HOPX in primary CRC, and findings obtained from basic research on the role of HPOX in cancer not including sarcoma, HOPX is likely implicated in the suppression of metastasis of CRC.

## 5. Downstream Gene of *HOPX* in CRC

**Figure 4.**
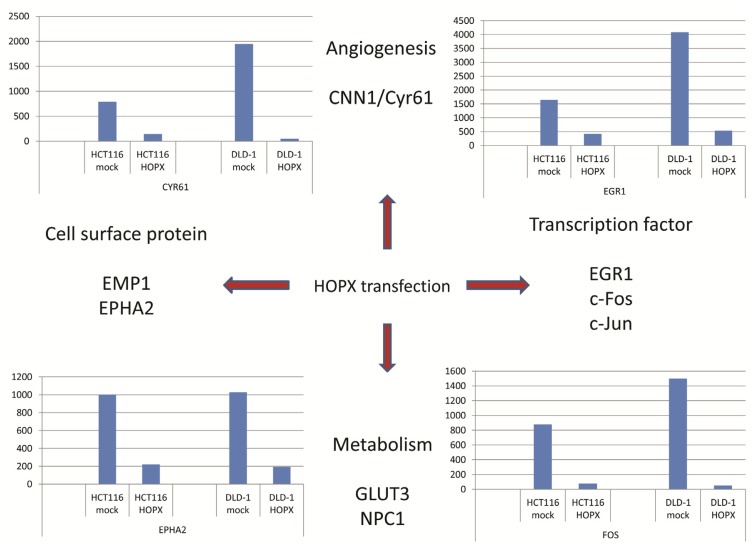
DNA microarray identified downstream targets of HOPX. Cell surface proteins such as EphA2 and EMP1, angiongenic growth factor of Cyr61, transcriptional factor such as c-Fos, EGR1, and metabolism-related genes like *GLUT3* and *NPC1* were tabulated.

**Figure 5.**
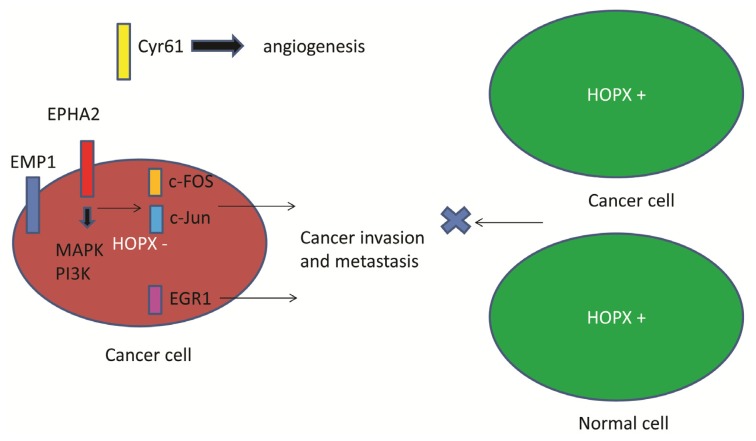
Model depicting HOPX-silenced cancer cells (**left red cell**) to induce EphA2, EMP1, c-fos, EGR1, and Cyr61, and result in metastatic formation. Transcription factors AP-1 and EGR1 may further induce cancer invasion and metastasis putatively through induction of matrix metalloproteinases and angiogenic growth factors. On the other hand, cancer cells with HOPX expression (**right green cell**) suppress such oncogenic molecules, and result in attenuation of invasive and metastatic properties like normal cells (**right green cell**). Green cells represent attenuation of invasive and metastatic properties against red cells that exhibited highly invasive and metastatic potential.

Since HOPX is involved in epigenetic regulation of differentiation-associated genes (Figure 2), the downstream targets of HOPX may include critical onco-proteins. However, there have been few reports describing downstream genes of *HOPX* in human cancers [12,26]. In CRC, expression microarray revealed that HOPX down-regulated oncoproteins. Cyr61, EMP1, EphA2, c-Fos, c-Jun, EGR1, and GLUT3 were identified as candidate genes [26] (Figure 4). Of these, c-Fos has been repeatedly described as a HOPX downstream gene in non-colonic cells such as endometrial cancer [29] and regulatory T cells (Treg) [10].

c-Fos is a component of AP-1 transcriptional factor and forms a heterodimer with c-Jun. HOPX-sufficient iT(reg) cells downregulated expression of the transcription factor AP-1 complex and suppressed other T cells [10]. In human cancer cells such as HEC and MCF7, forced expression of HOPX resulted in a partial block in cell proliferation, *in vivo* tumorigenicity, and *c-fos* gene expression in response to 17 beta estradiol (E2) stimulation [29]. In these cancer cells, analysis of the serum response element (SRE) of *c-fos* gene promoter showed that the effect of HOPX expression is associated with inhibition of E(2)-induced c-fos activation through the serum response factor (SRF) motif. These findings suggest that AP-1 is a critical downstream protein of HOPX through SRF. Transcription factor-binding sites of AP-1 are frequently recognized in the promoter region of cancer metastasis-associated genes such as matrix metalloproteinases [34,35] and tumor growth factors/receptors [36] (Figure 5). Consequently, such transcription factors could be decisive therapeutic targets in CRC. EGR-1 is another transcription factor downstream of HOPX that controls cancer progression through induction of IGF-II, [37], PDGF [38], and TGF-beta [39].

Tyrosine kinases such as EphA2 or EMP1 could be very interesting targets in cancer therapy because these cell surface molecules are more appropriate therapeutic targets than transcription factors. EphA2 is over-expressed in patients with HOPX hypermethylation [26]. Recent studies demonstrated that EphA2 is over-expressed in human cancers, and that EphA2 increases tumor invasion and survival, including of patients with CRC [40,41]. Thus, an EphA2 receptor antagonist, such as a specific tyrosine kinase inhibitor (in the form of an antibody, small molecule, peptide, or siRNA), or an antibody-drug conjugate that targets the EphA2 receptor, could be the basis for a novel targeted antineoplastic therapy [42–45]. EMP1 is an adhesion molecule that has been correlated with a lack of complete or partial response to gefitinib in lung cancer patient samples, as well as clinical progression to secondary gefitinib resistance. These findings suggest probable cross-talk between EMP1 and the EGFR signaling pathway [46]. EGFR could be an optimal target of CRC. EGFR antibodies (cetuximab and panitumumab) are active against CRC with no *K-ras* mutation [47,48], suggesting that EMP-1 holds promise as a predictive biomarker in antibody therapy.

In CRC, abundant expression of Cyr61 is found in patients with HOPX hypermethylation (Figure 4d) [26]. Cyr61/CCN1 is a matricellular protein and a member of the CCN family of growth factors. It is a critical downstream contributor to sonic hedgehog (SHh) that influences the pro-angiogenic tumor microenvironment [49]. CCN1-induced activation of SHh signaling may be necessary for CCN1-dependent *in vitro* cancer cell migration and tumorigenicity of cancer stem cells in a xenograft in nude mice [50].

Importantly, HOPX is likely to suppress independent oncogenic pathways, so multi-pathway inhibition may be required to control CRC with HOPX hypermethylation and silenced expression (Figure 5).

## 6. Quiescent Stem Cell (+4) and HOPX in Colon Goblet Cells

**Figure 6.**
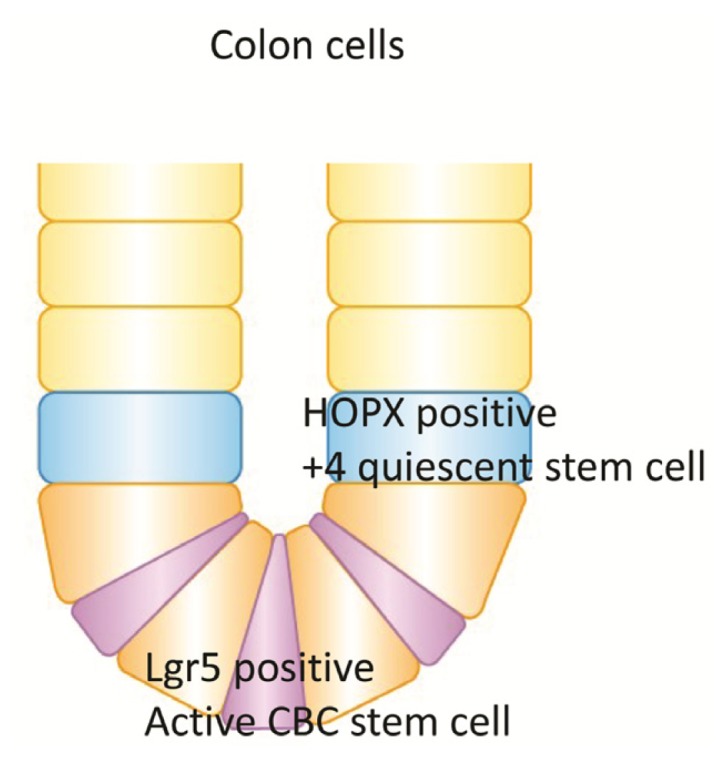
HOPX is a specific marker of the quiescent stem cells (+4), while Lgr-5 is a specific marker of the active stem cells (CBC) of the colon goblets.

In colonic mucosa, cells in the +4 niche are slow-cycling and label-retaining, whereas a different stem cell niche located at the crypt base is occupied by crypt base columnar (CBC) cells (Figure 6). It was recently reported that HOPX is a specific marker of quiescent stem cells (+4), while Lgr-5 is a specific marker of active stem cells in the colon mucosa [13,51]. CBCs are distinct from +4 cells, although both give rise to all intestinal epithelial lineages. Takeda *et al*. demonstrated that HOPX-expressing cells give rise to CBCs and all mature intestinal epithelial lineages. Conversely, CBCs can give rise to +4 HOPX-positive cells [13]. These findings demonstrate a bidirectional lineage relationship between active and quiescent stem cells in their niches, although it is unknown whether HOPX is epigenetically regulated or not in the colon stem cells.

In CRC, overexpression of LGR5 was significantly associated with expression of c-MYC, p21CIP1/WAF1/CDKN1A, and GLS, and inversely associated with miR-23a/b [52]. Immunohistochemical analysis indicated that Lgr5 may be embedded in benign adenomas, localized at the tumor-host interface, and detectable over a broad area in established tumors. A high level of LGR5 expression was associated with poor prognosis for CRC cancer patients who were curatively resected. Hence, the aggressiveness of CRC may be determined by the composite proportion of stem cells from which the cancer is generated.

## 7. Conclusions

In this article, we reviewed the current understanding of the relationship between HOPX and CRC. HOPX was initially reported as a differentiation marker of various tissues, and is expressed in normal organ tissues. In colon, HOPX is a quiescent colon stem cell marker and is expressed in differentiated colonic mucosa. The expression of HOPX is marked suppressed in a subset of cancers, mainly in an epigenetic manner. CRC may include separate entities which are differentially characterized by HOPX expression from a prognostic point of view. HOPX downstream targets were identified in CRC cell lines and primary CRC and could hold promise as candidates for therapeutic targets of CRC such as EphA2 or AP-1. Further analysis would elucidate and confirm the precise role of such protein molecules in CRC progression and their possible therapeutic value.

## Figures and Tables

**Table 1.** HOPX and human primary cancer.

Cancer Kinds	Histology	HOPX expression	DNA methylation	Prognostic relevance	References	Published year
Lung	SCC	Reduced in cancer	Not assessed	Not assessed	[27]	2003
	Adeno	Reduced in cancer	Not assessed	Yes	[12]	2013
Placenta	Tropho	Reduced in cancer	Not assessed	Not assessed	[28]	2003
Head and Neck	SCC	Reduced in cancer	Not assessed	Not assessed	[25]	2004
Esophagus	SCC	Reduced in cancer	Cancer-prone	Yes	[23]	2008
Uterine	Endo	Reduced in cancer	Cancer-prone	Not assessed	[29]	2009
Stomach	Adeno	Reduced in cancer	Cancer-prone	Yes	[20]	2010
Colon/Rectum	Adeno	Not assessed	Cancer-prone	Not assessed	[30]	2011
	Adeno	Reduced in cancer	Cancer-prone	Yes	[26]	2012
Pancreas	Adeno	Reduced in cancer	Cancer-prone	No	[21]	2012

SCC, squamous cell carcinoma; Adeno, adenocarcinoma; Tropho, trophoblast; Endo, endometrial carcinoma.

**Table 2.** HOPX and tumor suppressor function in human cancer cells.

Cancer Kinds	*In vivo* tumorigenesis	*In vitro* tumorigenesis	Proliferaion	Apoptosis	Invasion	Angiogenesis	Mets in animial	Ref.	Published year
Placenta	Yes	Not assessed	Not assessed	Not assessed	Not assessed	Not assessed	Not assessed	[28]	2003
Esophagus	Not assessed	Yes	Not assessed	Not assessed	Not assessed	Not assessed	Not assessed	[23]	2008
Head and Neck	Not assessed	Yes	Not assessed	Not assessed	Not assessed	Not assessed	Not assessed	[23]	2008
Lung	Yes	Yes	Yes	Not assessed	Not assessed	Not assessed	Not assessed	[19]	2007
	Not assessed	Not assessed	No	No	Yes	Not assessed	Yes	[12]	2013
Uterine	Yes	Not assessed	Yes	Not assessed	Not assessed	Not assessed	Not assessed	[28]	2009
Breast	Yes	Not assessed	Yes	Not assessed	Not assessed	Not assessed	Not assessed	[29]	2009
Stomach	Not assessed	Yes	Yes	Yes	Yes	Not assessed	Not assessed	[20]	2010
Colon/Rectum	Yes	Yes	Yes	Yes	Yes	Yes	Not assessed	[26]	2012
Pancreas	Not assessed	Yes	Yes	Yes	Yes	Not assessed	Not assessed	[21]	2012

SCC, squamous cell carcinoma; Adeno, adenocarcinoma; Tropho, trophoblast; Endo, endometrial carcinoma; Mets, metastasis.
